# REVIEW OF TECHNOLOGY-BASED INTERVENTIONS ADDRESSING SOCIAL DISCONNECTION AMONG DEMENTIA CAREGIVERS

**DOI:** 10.1093/geroni/igac059.267

**Published:** 2022-12-20

**Authors:** Weiyu Mao, Xiang Qi, Iris Chi, Bei Wu

**Affiliations:** University of Nevada, Reno, Reno, Nevada, United States; New York University, New York City, New York, United States; University of Southern California, Los Angeles, California, United States; New York University, New York, New York, United States

## Abstract

Social disconnection is a major public health concern. Informal dementia caregivers are particularly vulnerable to social isolation and loneliness, as the majority are older adults and at elevated risk of adversity. Technology-based interventions could offer accessible, affordable, and convenient solutions. Using a scoping review approach, we aimed to identify and synthesize existing technology-based interventions to address social isolation and loneliness among informal dementia caregivers. In a systematic search across six databases within the last ten years, ten eligible studies were included. The intervention type, format, and duration varied widely. The results suggested that technology-based interventions could reduce feelings of loneliness and improve caregiver well-being. The findings offer opportunities to use technology to reduce or prevent social isolation and loneliness. Investigating the role of technology in interventions addressing social isolation and loneliness among informal dementia caregivers could potentially overcome barriers to low uptake of services and improve sustainability of the interventions.

